# Electrical cream separator coupled with vacuum filtration for the purification of eimerian oocysts and trichostronglyid eggs

**DOI:** 10.1038/srep43346

**Published:** 2017-02-24

**Authors:** Saeed El-Ashram, Xun Suo

**Affiliations:** 1 State Key Laboratory for Agrobiotechnology & College of Veterinary Medicine, China Agricultural University, Beijing 100193, China; 2 National Animal Protozoa Laboratory & College of Veterinary Medicine, China Agricultural University, Beijing 100193, China; 3 Key Laboratory of Animal Epidemiology and Zoonosis of Ministry of Agriculture, Beijing 100193, China; 4 Faculty of Science, Kafr El-Sheikh University, Kafr El-Sheikh, Egypt

## Abstract

Several methods have been proposed for separation of eimerian oocysts and trichostronglyid eggs from extraneous debris; however, these methods have been considered to be still inconvenient in terms of time and wide-ranging applications. We describe herein an alternative way using the combination of electrical cream separator and vacuum filtration for harvesting and purifying eimerian oocysts and haemonchine eggs on large-scale applications with approximately 81% and 92% recovery rates for oocysts and nematode eggs obtained from avian and ovine faeces, correspondingly. The sporulation percentages as a measure of viability in the harvested oocysts and eggs from dry faecal materials are nearly 68% and 74%, respectively, and 12 liters of faecal suspension can be processed in approximately 7.5 min. The mode of separation in terms of costs (i.e. simple laboratory equipments and comparably cheap reagents) and benefits renders the reported procedure an appropriate pursuit to harvest and purify parasite oocysts and eggs on a large scale in the shortest duration from diverse volumes of environmental samples compared to the modified traditional sucrose gradient, which can be employed on a small scale.

Many studies have been devoted to coccidiosis owing to significant economic losses to the poultry and many other domestic livestock industries throughout the world. Coccidiosis is a severe infection caused by the apicomplexan parasite of genus *Eimeria* in a host and predilection site specific manner^1^,^2^,^3^,^4^. Furthermore, among the gastrointestinal parasites that cause losses to the farming industry, for example, *Ostertagia, Trichostrongylus, Nematodirus and Cooperia, Haemonchus contortus* or the barber’s pole worm is the predominant nematodes that infect small ruminants^5^.

Coprologic examinations have long been conceded as an effective way for parasite identification that animal and human contracted with them. The most commonly employed technique in veterinary medicine, medical and clinical laboratory for separation of oocysts and eggs is the faecal floatation test. The technique depends on the differences in the specific gravity of the oocysts and eggs, the fluid floatation medium and debris. In other words, the differential density exists between them. Centrifugal flotation methods are clearly superior to non-centrifugation flotation procedures in terms of rapidity and efficiency.

A considerable amount of literature has been published on eimerian oocyst and nematode egg separation from faecal materials. Techniques for the concentration and purification of oocysts and eggs from faecal samples include saturated salt solution flotation, sucrose density, zinc sulfate, and Percoll discontinuous density gradient centrifugation. Sucrose gradient ultracentrifugation has been exploited to separate subcellular fractions of *Eimeria tenella* sporozoites^6^. More recent attention has focused on the isolation of the endocytic organelle from macrophages by sucrose gradient^7^. In 1987, Arrowood and Sterling^8^ published a paper in which they employed discontinuous sucrose gradients and isopycnic Percoll gradients to isolate *Cryptosporidium* oocysts and sporozoites. Early example of research has examined the relationship between discontinuous sucrose gradient and viability of *Cryptosporidium* sp. oocysts, and reported this method as a simple and rapid mean to obtain viable oocysts^9^. In a study which set out to purify *Toxplasma gondii* tacyzoites, Garberi *et al*.^10^ found that tachyzoites obtained by two consecutive discontinuous sucrose gradient separation maintained its biological activity.

On the other hand, the application of ‘salt-sugar’ isolation method to purify gastro-intestinal nematode eggs in such a way that allows automated analysis and high-throughput screening of nematode egg digital image preparations has been reported by^11^. Similarly, the combination of a low-density salt solution for flotation and centrifugation, and sucrose flotation and precipitation has been employed to obtain purified nematode eggs^12^. A recent study by^13^ has employed two-step method, sucrose flotation followed by density gradient centrifugation exploiting the lymphocyte separation medium (LSM) to produce highly-purified nematode eggs. Sharma *et al*.^14^ reported a method involving density gradient centrifugation to obtain pure oocyst suspensions free of foreign material. All the aforementioned floatation methods are sufficient in varying degrees for recovery of small quantities of eimerian oocysts and nematode eggs. On the other hand, large-scale method for separating oocysts involving sedimentation of heavier particles from faecal suspension, collection of oocysts from the supernatant, repeated washing and settling in potassium dichromate (K_2_Cr_2_O_7_) solution. This method; however, results in continuous loss of oocysts (i.e. extensive washing of oocysts) and large volumes of waste water^15^. Similarly, Eckert *et al*.^16^ have lengthily described typical laboratory methods involving screening, sedimentation and gravitational salt flotation for oocyst isolation from faeces. This technique is for the concentration and purification of oocysts from large quantities of faecal samples, necessitates long periods of time and some concerns about the efficiency of oocyst and egg recovery rates (unpublished data).

A number of factors need to be considered when the large-scale collection and purification of oocysts and eggs of parasitic infections from faecal materials, for instance, inoculum, feeding status of animals at the time of inoculation, age of animal, collection period, feeding status during collection, collection medium, homogenization, floatation, sieving, washing, sporulation, hypochlorite treatment and further purification^17^. Faecal dilution^18^ is an important factor in the large-scale extraction of eimerian oocysts and nematode eggs from faeces. For this reason, different dilutions, such as 1:5, 1:7.5, 1:9, 1:10, 1:12.5, 1:15 and 1:20 should be tested to determine the optimal faecal dilution. A series of dilutions of pooled faecal samples from chicken and sheep containing defined amounts of the spike-in oocysts and eggs, respectively was used to determine the optimal faecal dilution ratio. In addition, the oocyst and egg detection limit (oocysts and eggs per gram of faeces) was determined by spiking faecal samples with decreasing numbers of oocysts and eggs. Faecal-spiking was also employed to determine the influence of different chicken and rabbit pure food used alone or in combination with fresh chicken and rabbit faecal materials on the recovery rate owing to the feeding behavior of the animals could result in mixing animal excreta with food materials in a varying degree.

In any coccidia and helminth parasite research program, it is indispensable to extract and purify oocysts and eggs from faeces for a wide range of scientific and industrial processes; the larger yields and the higher degree of oocyst and egg purity required will depend on the subsequent material usage. Therefore, the overall concern in all conventional methods has been to develop an efficient procedure for viable oocyst and egg isolation, which can be applied on a large scale in the shortest duration. Driven by this need, we proposed here a simple two-step procedure for obtaining highly purified oocysts and nematode eggs. The combination of the cream separator and filtration pump was exploited to harvest parasite oocysts and eggs of a required degree of purity and viability in large-scale, with the disbursement of the minimum amount of physical effort, time, animals, chemicals and glassware. For comparison purposes, a modified discontinuous (step-gradients) sucrose gradient method was employed for the isolation of eimerian oocysts and haemonchine eggs.

## Materials and Methods

### Ethics Statement

Animal experiments were conducted in accordance with the guidelines of Beijing the Municipality on the Review of Welfare and Ethics of Laboratory Animals approved by the Beijing Municipality Administration Office of Laboratory Animals (BAOLA), and under the protocol (CAU-AEC-2010–0603) approved by the China Agricultural University Animal Ethics Committee. All experimental procedures were also approved by the Institutional Animal Care and Committee of China Agricultural University (The certificate of Beijing Laboratory Animal employee, ID: 15883).

### Eimerian oocyst propagation

Two-week-old specific pathogen free (SPF) Arbor Acres (AA) broiler chicks were purchased from Beijing Arbor Acres Poultry Breeding Co., Ltd. They were housed in isolators and fed with a pathogen-free diet and water. The climatic conditions, lighting program, and chicken fodder and water were manually-operated and the chicks were cared for in agreement with the approved guidelines of the Institutional Animal Care and Committee of China Agricultural University. Sporulated oocysts (2.5 × 10^3^) were inoculated into broiler chicks by oral gavage, and faeces were collected at 5–9 days post-inoculation according to eimerian species and the experimental requirements^19^,^20^,^21^. The collected oocysts of *Eimeria mitis* were used to spike other oocyst-free chicken faeces.

### Seed-and- recovery experiment for validation of the current method by using different irrevelant matrices

The use of *E. mitis oocysts* recovered from freshly experimental infection to spike different chicken and rabbit pure food used alone or in combination with fresh chicken and rabbit faecal materials in a ratio of 1 (chicken food) to 5 (chicken or rabbit faecal materials). Furthermore, the ratio of 1 (rabbit faecal material) to 5 (rabbit food) was tested. Faeces from experimentally infected animals were collected from chicken and rabbit. Each sample was mixed with a known amount of freshly collected oocysts. The seeded matrices were homogenized with saturated NaCl solution at a 1 (80 g):12.5 (920 ml saturated NaCl solution) ratio employing a suitable laboratory mixer until the mixture was homogeneous as described below.

### Trichostronglyid egg preparation

Two-month-old sheep (*Ovis aries*) that were raised under confined living conditions to avoid worm exposure were moved from a local farmer in Jin Zhan village, Chaoyang, Beijing, China with an average liveweight of 25.02 kg ± 2.2 kg. Faecal materials and blood were collected for parasitological, haemato-biochemical, immunological analyses (unpublished data) to confirm absence of infection. Upon arrival in China Agricultural University facilities, all sheep were tagged (each sheep was assigned an arbitrary number for sample identification purposes), weighed, and then treated with double doses of ivermectin (Shijiazhuang Fengqiang Animal Pharmaceutical Co., Ltd) and a single dose of levamisole (Hebei New Century Pharmaceutical Co., Ltd). The sheep were acclimatized for four weeks before infection experiments initiated. The animals received a diet containing forage (grass silage): concentrate (barley, maize, wheat, wheat bran and bruised soya) 1:1 ad libitum. Sheep were exposed to a 12 h/12 h light/darkness regimen at mean internal relative humidity (23.4% ± 2.5%) and temperature (19.28 °C ± 0.94 °C) within the dwelling facility. Infective 3^th^ stage larvae (L_3_) of *H. contortus* were kindly provided by Professor Dr. Hu from State Key Laboratory of Agricultural Microbiology, College of Veterinary Medicine, Huazhong Agricultural University, Wuhan 430070, Hubei, China. A total of three; 3-month-old sheep were orally infected with a single dose of 5,000 *H. contortus* infective 3^rd^-stage larvae (L_3_) and maintained for 50-day post-infection. Faeces were collected at 14–50 day post-inoculation according to the experimental requirements.

### Purification methods

#### Oocyst and egg concentration by the electrical cream separator machine

**Figure 1:**
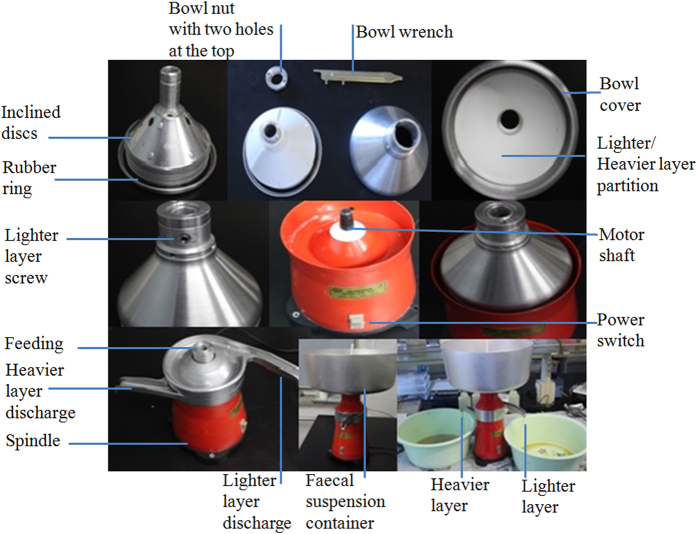
Different parts of electrical cream separator.

**Figure 2:**
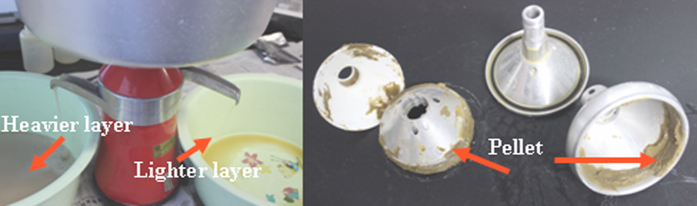
Three phase electrical cream separator (lighter, heavier and pellet).

**Figure 3:**
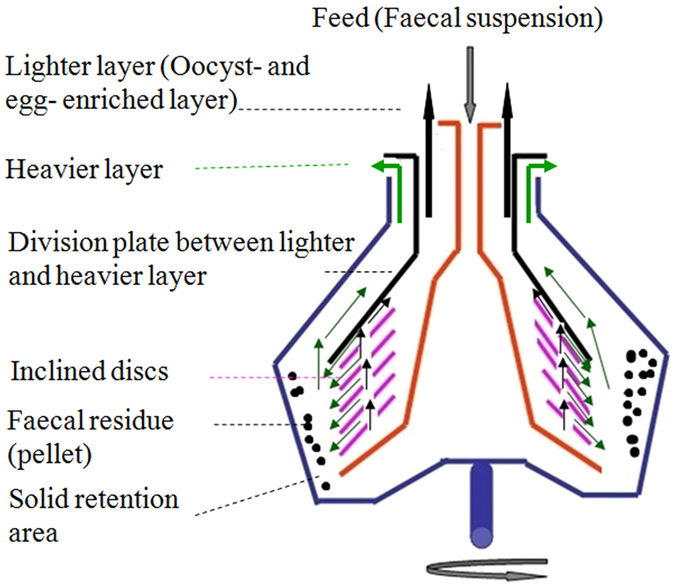
Schematic drawing of the centrifugal separation process.

Cream separator machine (MOTOP C14-100, Ukraine) can be used throughout the separation process, which can be looked at as the three-phase separator machine (Figs 1 and 2). The cream separator machine consists of a bowl spinning around a vertical axis, as illustrated in Fig. 3. Faecal suspensions are introduced through the feed inlet under centrifugal force. The heavier liquid and faecal residue pass to the distant regions of the bowl, while the lighter layer (oocyst- and egg- enriched layer) moves to the center. The conical plates (inclined discs) are arranged as shown in Fig. 3 to give smoother flow and better separation. For maximal recovery rate, the heavier layer can be reprocessed again, and the pellet can be collected and blended with the fluid floatation medium for 1 min to separate the remaining number of the parasite oocysts and eggs. The manual cleaning and discharge of faecal residue from the conical bowl can be carried out between runs. However, the fully automatic centrifugal cream separator machine with mechanical washing and discharge of faecal residue (conical bowl with nozzle) can process unlimited amount of faecal materials (i.e. continuous feeding of faecal suspension and ceaseless discharge of faecal residue).

### Cream separator machine assembling procedure

The cream separator machine set up can be divided into ten steps. These are:

Step 1: Put the rubber ring into the slot, and mount the inclined discs sequentially.

Step 2. Cover the discs with the separating plastic plate and then with bowl cover.

Step 3. Screw the nut with a hand and then tighten it with a bowl wrench by inserting two jets of the bowl wrench into two holes of the nut.

Step 4. Install the assembled drum onto the spindle, and then install the following parts: Heavier layer discharge, lighter layer discharge and cream separator container.

Step 5: Close the cork of the cream separator container.

▶Critical step: The installation area shall be horizontal and adjusted with a level gauge.

Step 6: Turn on cream separator and wait 30 seconds until it reaches its maximum speed.

Step 7: Pour the sieved faecal suspension into the cream separator container.

Step 8: Open the cork of the cream separator container.

Step 9: Collect the lighter and heavier layers.

▶Critical step: The bowl disassembly is achieved in reverse order of its assembly.

Step 10: Boil the bowl components for 10 min after washing to switch between different eimerian oocyst and haemonchine egg species.

### Adjustment of oocyst- and egg- enriched lighter layer volume

Step 1. Adjust the lighter layer volume by turning the lighter layer regulating screw at the upper part of the separating plate using the lighter layer regulating key.

**Figure 4:**
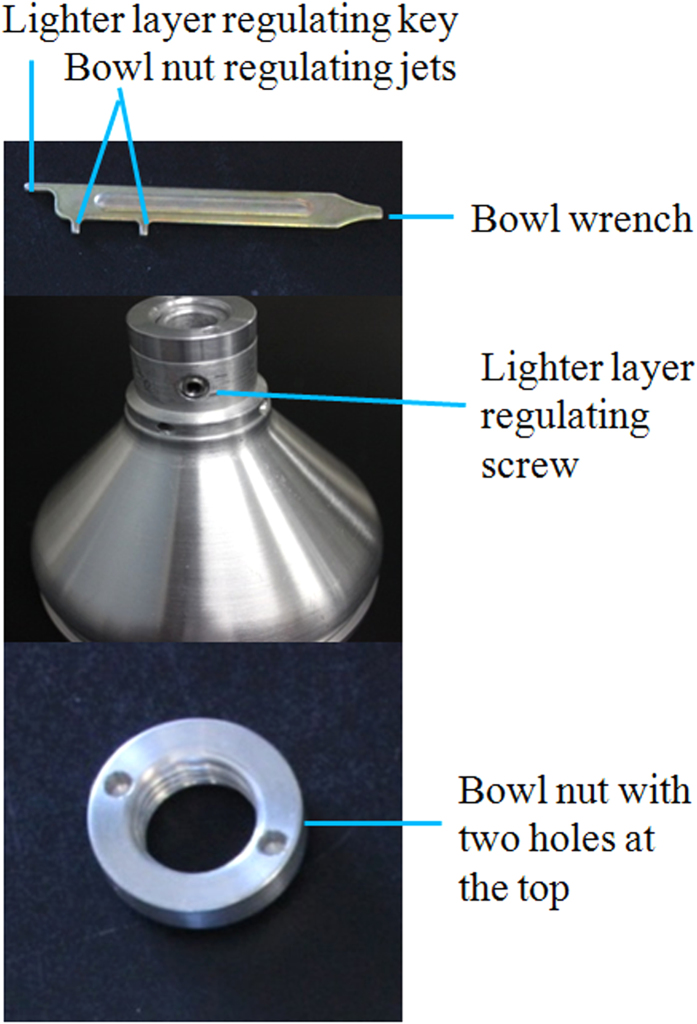
Cream separator components responsible for regulation of oocyst- and egg- enriched lighter layer volume.

Step 2. Turn the lighter layer regulating screw clockwise for smaller volume, and counterclockwise for larger volume (Fig. 4).

▶Critical step: it is desirable to drive the lighter layer regulating screw out for oocyst- and egg- enriched lighter layer.

### Procedural steps for coping with the egg-/oocyst- containing faecal material

The oocyst-/egg- containing faecal material was mixed with saturated solution of sodium chloride (NaCl) in the ratio of 80 g of faecal material diluted and rehydrated with 920 ml salt solution. After being blended for 1 min (i.e. to avoid trapping of the oocysts and eggs) and squeezed through four layers of cheese cloth (the residue on the cheese cloth is again diluted and resqueezed), the oocyst-/egg- containing faecal material is placed in the electrical cream separator container and centrifuged at a high speed (approximately 10000 rpm) for 5 min.

### Vacuum filtration for residual faecal material removal

**Figure 5:**
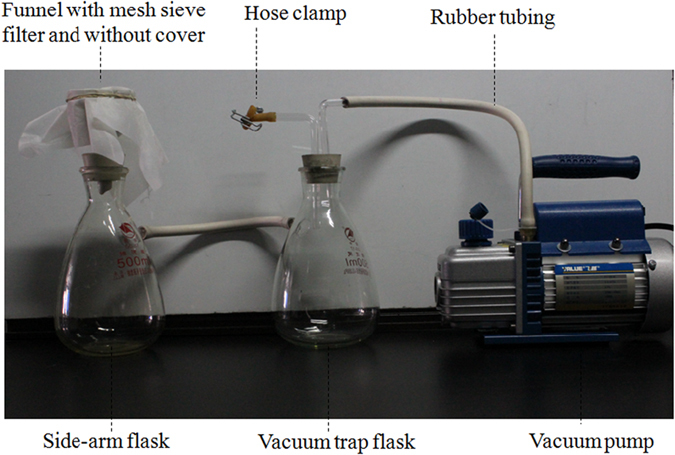
Set up for filtration assemblage to remove the residual faecal material (fine debris) and microbial contaminants.

Filtration is the passage of the lighter layer through a filter with pores small enough to remain oocysts and eggs and to eliminate fine debris. With respect to mesh sieve pore sizes, mesh sieve filters (ELKO Filtering Co, LLC, USA) ranged from 5 μm (*E. mitis*) to 35 μm (*H. contortus*) were used throughout this study. A 10 μm mesh sieve filter is the correct size for *Eimeria maxima, Eimeria brunetti* and *Eimeria praecox* and for other chicken eimerian parasites the 5 μm mesh sieve filter is applied to avoid leakage. The oocysts and eggs accumulated on the mesh sieve filter were washed with distilled water and collected using 2.5% potassium dichromate and distilled water, respectively for parasite development. As shown in Fig. 5, the mesh sieve filter is present at the top of ceramic funnel and the flask with one side-arm attached to rubber tube connected with another vacuum trap flask. The latter is connected with vacuum pump, which has an advantage over the ordinary gravitational force.

### Cream separator machine coupled with vacuum filtration procedure

The whole separation process for eimerian oocyst of chicken and trichostrongylid egg of sheep employing cream separator machine can be listed as follows:

Step 1: Mix 80 g of faecal material with 980 ml of saturated salt solution (1:12.5).

▶Critical step: keep some salt solution from step 1 to add them to the residue (step 4) on the cheese cloth and resqueeze again.

Step 2: Homogenize with a laboratory mixer until the mixture is homogeneous (i.e. approximately 1 min).

Step 3: Squeeze homogenate through four layers of wet cheese cloth (i.e. immersed in saturated salt solution) to remove large debris.

▶Critical step: Soak the cheese cloth in saturated salt solution to avoid trapping of oocysts and eggs.

Step 4: Repeat step 3 using the residue on the cheese cloth and resqueeze again.

Step 5: Mix the filtrate from step 1 and step 3.

Step 6: Apply filtrate to the faecal suspension container of cream separator machine, when it reached its maximum speed.

Step 7: Collect the lighter, heavier layers and pellet in separate containers.

▶Critical step: Reapply the heavier layer as described above (step 6) and the pellet (faecal residue) as above (step 1) to increase the recovery of the oocysts and eggs from the faeces.

Step 8: Apply cream separator machine-purified oocysts and eggs (lighter layers) to vacuum filtration for salt and fine debris removal using a suitable mesh sieve filter and plain tap water for 2 min.

Step 9: Collect the accumulated oocysts and eggs using plastic pasteur pipette and the suitable incubation solutions (2.5% potassium dichromate for eimerian oocysts and distilled water for trichostronglid eggs) for subsequent processes.

▶Critical step: Bleach the collected oocysts in 4 to 5 volumes of ice-cold household bleach (sodium hypochlorite, ~5% w/v aqueous) and placed on ice for 10 min with occasional shaking. Then wash thoroughly as described above (step 8) under aseptic conditions. Similarly, sterilize the accumulated eggs by washing twice with 2% sodium hypochlorite for 20 s each followed by five times with sterile distilled water under disinfected conditions. Conduct this critical step for metabolic studies of oocysts/eggs, sporozoites/larvae used for cell or tissue culture inoculation or studying the microbial community associated with nematode eggs (unpublished data).

**Figure 6:**
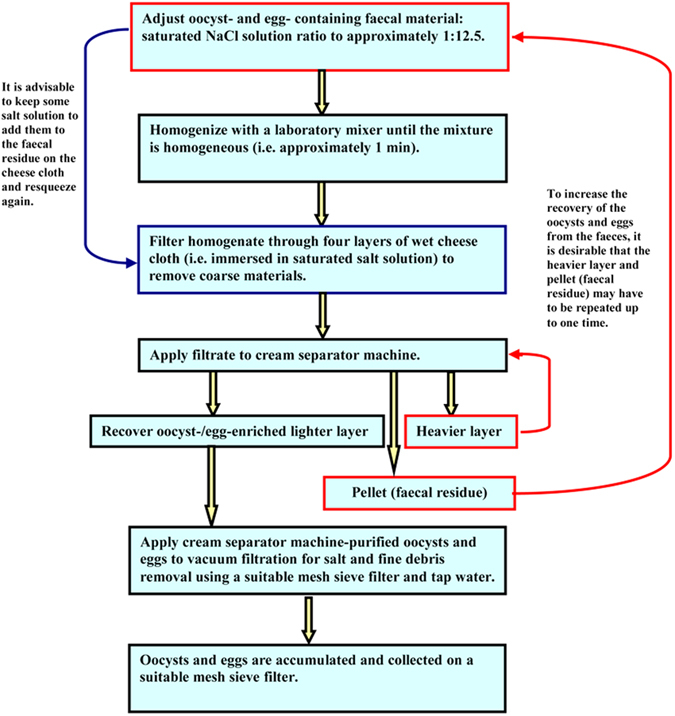
Flow chart summarizing the concentration and purification of eimerian oocysts and trichostrongylid eggs.

A Flow chart recapping the concentration and purification of eimerian oocysts and trichostrongylid eggs are set out in Fig. 6.

### Oocyst and egg separation on a discontinuous sucrose gradient

**Figure 7:**
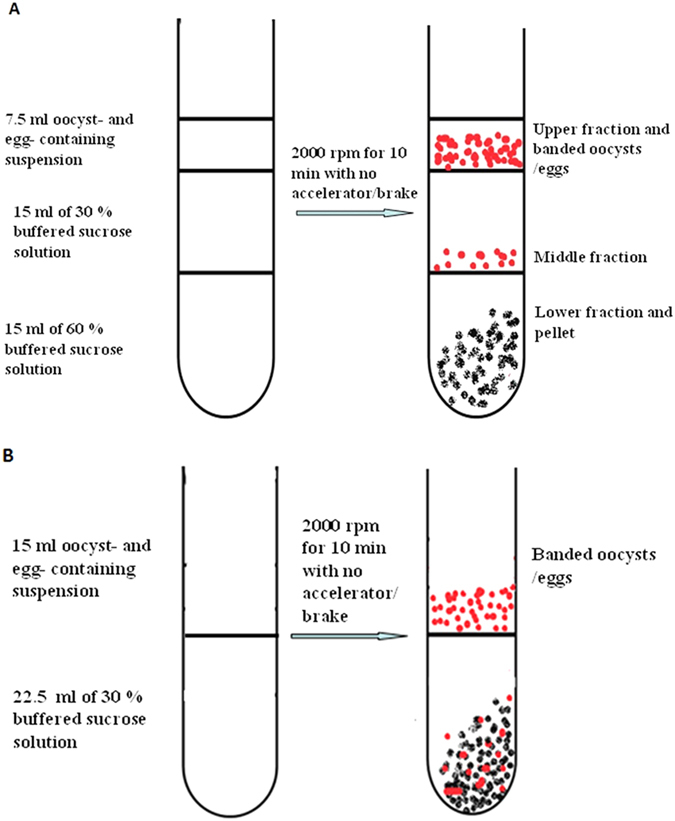
Sucrose density gradient centrifugation. Conventional sucrose gradient set up (**A**) and alternative sucrose set up (**B**).

**Figure 8:**
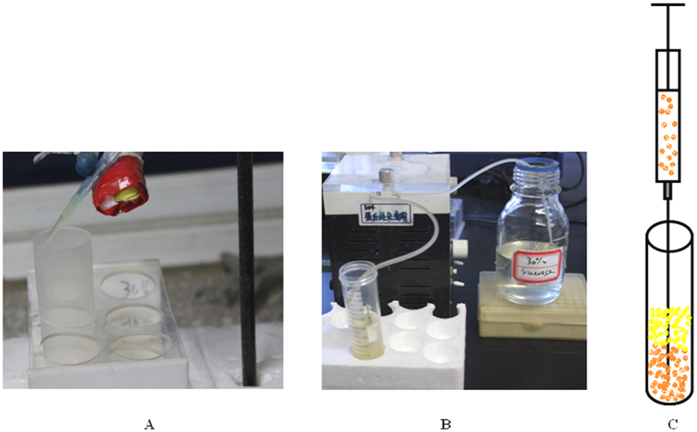
Setup for sucrose gradient. (**A**) Over-layering preparation using yellow pipettor tip and blue pipettor tip, which are held substantial by a clamp stand. (**B**) Over-layering preparation using the peristaltic pump. (**C**) Under-layering preparation using the syringe and needle.

Oocysts and eggs were recovered according to^10^ with major modifications as follows: The buffered sucrose solution (PBS-Ca buffer, PBS, PH 7.4 plus 1 mM CaCl_2_) is an ideal density gradient material owing to low cost, inertness, stability, high solubility, and non-toxic nature. Furthermore, removal of fine debris and sucrose from separated oocysts and eggs is best achieved by repeated washings with distilled water or a suitable buffer under vacuum filtration. To decrease the osmotic strength with sucrose solutions and the effect of the centrifugal force, the centrifugal speed and time (i.e. 2000 rpm for 10 min) are reduced to maintain high oocyst and egg viabilities. Preformed gradients of the discontinuous sucrose are prepared by carefully layering two consecutive solutions (15 ml each) of different density (i.e. 60% and 30%) on top of each other in the 50 ml centrifuge tube with a concrete shift in density (a step) between each (Fig. 7A). A small amount of phenol red was added to the 30% sucrose solution to easily discriminate between the gradient layers. Preformed gradients of discontinuous sucrose used for oocyst and egg separation can be prepared using over-layering (Fig. 8A and B) and under-layering techniques (Fig. 8C). The gradient is prepared by layering progressively less dense 30% sucrose solution upon 60% by slowly allowing it to run down inside the yellow (200 μl) pipettor tip, which is placed on the end of the blue (1000 μl) pipettor tip, and gravity will drain the solutions down the side of the tube steadily as shown in Fig. 8A. Other applications of the discontinuous sucrose solutions included the setup described below in Fig. 8B using the peristaltic pump (Peristaltic pump, HL-2B; Shanghai Jingke industrial co. LTD) and the under-layering technique (Fig. 8C) [a syringe with a large needle is employed to introduce the denser sucrose solution (i.e. 60%) under the lighter one (i.e. 30%)]. A 7.5 ml of oocyst- or egg- suspension (80 g of faecal material diluted and rehydrated with 980 tap water) after being blended for 1 min (i.e. to avoid trapping of the oocysts and eggs) and squeezed through four layers of cheese cloth (the residue on the cheese cloth is again diluted and resqueezed) is layered on top of the gradient as a narrow band (i.e. to avoid streaming) and centrifuged at a low speed. On centrifugation, the oocysts and eggs mainly banded at the interface between the two densities (i.e. lighter oocyst- and egg- containing suspension and denser sucrose solutions, 30%). As most of the oocysts/eggs are collected at the interface between the sample (oocyst- and egg-containing solutions) and the 30% sucrose, this one-step method involving sucrose step-density gradient centrifugation for concentration and recovery of eimerian oocysts and trichostronglyid eggs free from residual fecal materials could be simplified to have one continuous gradient (30%), which will produce the so-called concentrated oocysts and eggs on top of the 30% sucrose solution (Fig. 7B). This additional sucrose float is necessary to minimize faecal debris carried over the suitable mesh filter under vacuum for further purification and collection.

Oocysts and eggs isolated (enriched) by the sucrose method were subjected to a vacuum filtration through 5 μm (*E. mitis*) and 35 μm (*H. contortus*) nylon meshes for fine debris and sugar removal using thorough washings with plain tap water. The accumulated oocysts and eggs by filtration over the sieve were collected in 2.5% potassium dichromate (eimerian oocysts) and distilled water (haemonchine eggs), respectively. The numbers of oocysts and eggs recovered were adjusted by centrifugation at 3000 rpm for 5 min and addition of the required volume of the previous solutions.

### Recovery rate

The average oocyst recovery rate (as a percentage) was calculated by using the formula [(oocyst number recovered/(oocyst number experimentally detected or seeded)] × 100; however, the average egg recovery rate was computed by employing the sentential function [(egg number recovered/(egg number experimentally detected)] × 100. To ensure the procedure in use harvests reliable and reproducible estimates, the flotation time (i.e. the interval between loading the McMaster chamber and counting the oocysts/eggs) in the well-mixed oocyst/egg saturated NaCl suspension is 5 min at room temperature. In order to obtain accurate oocyst numbers using McMaster chamber^22^,^23^, several counts from different samples are performed.

### Viability assay

#### Eimerian oocyst sporulation

For sporulation, the freshly purified oocysts were transferred into 2.5% potassium dichromate solution (w/v, aqueous) in Erlenmeyer flasks covered with a perforated lid on a rotary shaker at 26 °C and 80% relative humidity for approximately 72 h^20^. Sporulation was confirmed by examining a drop from each aliquot under the microscope. The examined oocysts were considered to be sporulated when four sporocysts were apparently distinguishable in each oocyst. At least five hundred oocysts were checked in each aliquot, which was regarded as the satisfactory number for statistical analysis and the number of sporulated oocysts was recorded. The average viability rate (as a percentage of sporulation) was calculated by using the formula [(sporulated oocysts)/(total number of oocysts)] × 100.

### Trichostronglyid egg hatching

The extracted nematode eggs were washed on a 35 μm nylon filter with distilled water to remove fine debris and salt (cream separator machine) or sugar (modified sucrose gradient). The egg was adjusted to 500 eggs per well in 6-well cell culture plate containing distilled water. The plate was incubated for 7 days at room temperature to allow parasite development to the L_3_ larval-stage. The total number of L_3_ larvae was recorded. The average viability rate (as a percentage of hatching) was calculated by using the formula [(total number of L_3_ larvae)/(total number of eggs)] × 100.

### Optical microscopic observation

Optical microscopic observation was used for purity (qualitative) evaluation of oocyst and egg purification. More than 100 microscopic fields from different prepared slides of coccidian oocysts and nematode eggs were investigated before considering the oocysts, and eggs are pure without any faecal contaminants.

### Statistical analysis

A two-tailed *t*-test using Instat software (Graphpad Software, San Diego, Calif) was employed to determine statistical difference between recovery and viability rates, and required time of oocysts and eggs harvested by cream separator method and modified sucrose gradient method. Statistical analysis (a two-tailed *t*-test) was carried out to determine the superiority of 1:12.5 faecal dilution ratio over other faecal dilution ratios. A *P-* value of less than 0.05 was considered statistically significant.

## Results

The present study portrayed the use of discontinuous sucrose gradient and cream separator machine in conjunction with a vacuum pump for concentration and purification of eimerian oocysts and trichostrongylid eggs.

### Cream separator machine coupled with vacuum filtration for separation of oocysts and eggs

The efficiency of separation increased due to the inclined discs that reduced the distance to the bottom of the bowl. Therefore, the coarser materials will slide in the bottom of the bowl and more purified layers will be produced. The lighter layer (from the top outlet), which contains the parasite oocysts and eggs is less dense than the fluid floatation medium float and proceed in direction to the centers of the bowl and leave the drum (bowl) from the cream output under the centrifugal force within the rotating drum of the cream separator. The heavier layer, which contains the negligible value of the parasite oocysts and eggs proceeds over the outside of the bowl and reaches to the heavier layer outlet. The pellet (faecal residue), which contains coarse material and the leftover of the parasite oocysts and eggs is accumulated in the solid retention area.

### Optimal dilution ratio, and minimal and maximal recovery rate determination

**Figure 9:**
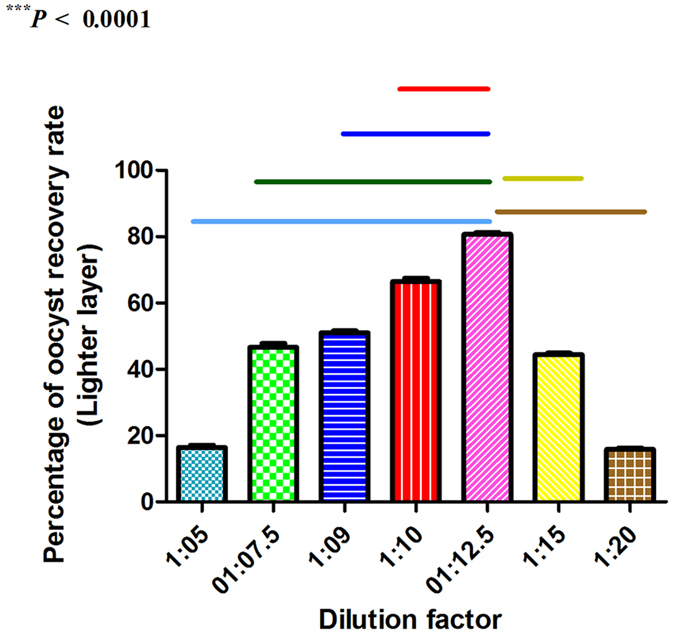
A graph showing the optimal faecal dilution ratio (1:12.5) compared with other dilution ratios. ***P < 0.0001.

Different combinations of faecal material and saturated saline solution were used to determine the optimal mixing ratio that yielded the maximum recovery rate (Table 1; Fig. 9). From these data, we can see that a 1 (80 g of faecal material):12.5 (920 ml of saturated saline solution) dilution factor resulted in the highest average recovery rates of oocysts [80.68% (SD, ±1.61%)] for the lighter layer (***P < 0.0001). Consistent with this dilution factor, the average recovery rates of nematode eggs were 91.91% (SD, ±1.35%) for the lighter layer. The lower detection limit per ml of faecal suspension was 0.9% (SD, ±0.74%) and 1.6% (SD, ±0.55%) for oocysts and eggs, respectively (Table 2).

### Effect of diverse extraneous matrices on oocysts’ recovery efficiency

Oocysts recovered from freshly experimental infection were used to seed different food and faecal material combinations. Table 3 summarizes the average recovery rate of eimerian oocysts (expressed as a percentage) from seeded pure and mixed combinations of diverse matrices using our current approach. Strikingly, the recoveries were adversely different.

### Viability assay

**Figure 10:**
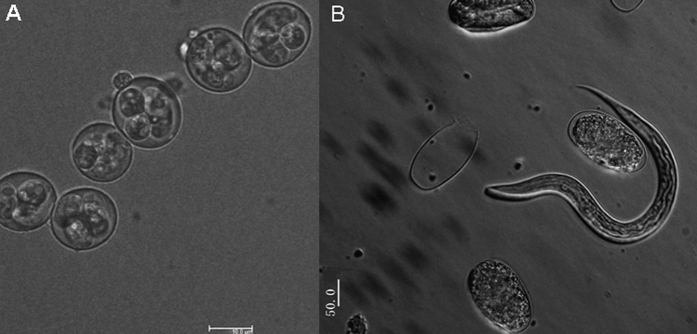
Photomicrographs of the viability assay. Atypical sporulated oocyst of *Eimeria mitis* sporulated for 72 h (**A**) and larval development process of *Haemonchus contortus* incubated for 24 h (**B**).

The most important feature of any oocyst- and egg- isolation method is maintenance of cell viability. An experiment was performed to determine the rate of hatchability of nematode eggs employing the current isolation method. The extracted nematode eggs were washed on a 35 μm nylon filter with distilled water to remove the salt. The egg was adjusted to 500 eggs per well in 6-well cell culture plate containing distilled water. The plate was incubated for 7 days at room temperature to allow parasite development to the L_3_ larval-stage. Interestingly, our results showed 73.74% (SD, ±7.61%) larvae hatching out of their eggs (Fig. 10B).

### Combined sucrose step-density gradient centrifugation- vacuum filtration methods for oocyst and egg isolation

#### Maximal and minimal recovery rates

It may be concluded from the present study that the peristaltic pump and under-layering technique are comparatively reliable techniques than over-layering techniques and may be recommended to use for reproducible sucrose gradient formation without disturbances of the gradient layers. An aqueous-based contamination and sugar removal could be performed on a suitable mesh filter using a vacuum pump under the above-mentioned conditions. Then, the average rate of recovered oocysts and eggs in the upper fraction was found to be 81.87% (SD, ±9.81%) and 92.32% (SD, ±3.13%), respectively. However, the average of the minimal detectable rate of eimerian oocysts and nematode eggs per ml of faecal suspension that could be detected was 1% (SD, ±1.03%) and 1.2% (SD, ±0.8 4%), correspondingly (Table 2).

### Viability assay

Under optimal conditions (26 °C, aeration, 2.5% K_2_Cr_2_O), freshly isolated *E. mitis* oocysts displayed a higher 68.06% (SD, ±1.9%) sporulation ability of oocysts within 72 h (Fig. 10A).

### Microscopic examination of concentrated and recovered coccidian oocysts and nematode eggs

Optical microscopic observation illustrated that the extracted oocysts and eggs were highly purified in both the discontinuous sucrose gradient- and cream separator machine- associated with a vacuum pump. Additionally, the isolated oocysts and eggs were free of bacteria [after washing with laundry bleach, sodium hypochlorite (NaOCl)] and faecal contaminants (unpublished data).

### Comparison of milk cream separator with that of the modified sucrose gradient

Overall, the cream separator machine gave slightly lower recovery rates (as a percentage), especially in the context of maximum yield and minimal detection limit than the discontinuous sucrose gradient; however, the differences between the oocyst and egg recovery rates were not significant (*p > *0.05). Moreover, the major advantages associated with the use of current method in comparison with the conventional methods, such a modified sucrose gradient can be seen from the data in Table 2. Although there is a high recovery rates of oocysts (~81%) and nematode eggs (~92%) obtained from avian and ovine fecal materials, respectively using the cream separator method, neither of them was significantly higher than that of the modified sucrose gradient (*p* > 0.05). Additionally, there were no significant differences between the viability rates from traditional sucrose gradient method compared with our current approach (*p* > 0.05). However, the indispensible manual dexterity was less with the cream separator method. Moreover, the required time to process 12 liters of faecal suspension (~7.2 min) (***p < 0.0001) was less than for the modified sucrose method (approximately 16.6 days).

## Discussion

Most procedures that have been delineated for concentrating and purifying eimerian oocysts and nematode eggs are designed to either identify the parasites in veterinary clinical specimens or isolate oocysts and eggs from a small volume of faeces from infected avian and ovine hosts. The present study describes a rapid method for the large-scale extraction, purification and cleaning of coccidial oocysts and trichostrongylid from faeces free of unwanted faecal and microbial contaminants. The isolation method is based on saturated NaCl centrifugal flotation technique of oocysts and eggs, followed by decontaminating, washing and accumulating on suitable nylon filters under electrical vacuum.

Floatation solution includes sugar and various salt solutions, such as saturated sodium chloride, sodium nitrate, magnesium sulfate and zinc sulfate. The choice of floatation solution has a substantial effect upon the sensitivity of the flotation procedures^24^. Advantages and disadvantages should be considered when selecting the fluid floatation medium to concentrate the parasite oocysts and eggs. By way of illustration, our data show how the mixing ratio between the faecal materials and the fluid floatation solution can influence the recovery rate. The oocyst- containing faecal materials were serially diluted using the most inexpensive and easiest floatation solution (table salt) to explore the correct mix between them. The most striking result to emerge from the data is that the ratio between the faecal material and floatation solution influences the oocyst recovery rate. The lesser the dilution ratio, the more collision between faecal material particles with each other than with the disc stack (inclined discs) resulted in minor recovery rate of the oocysts. Compared with the lower dilution ratio, the higher dilution ratio of the faecal material led to flowing away of the particles of faecal material and incomplete separation process. Different combinations of faecal material and saturated saline solution were used to determine the optimal dilution ratio that yielded the maximum recovery rate. On examination of the optimal dilution ratio, it was revealed that a 1:12.5 dilution factor had approximately 81% and 92% for oocysts and eggs, respectively. In this context, the influence of sample dilution on faecal egg counts based on the McMaster technique has been reported by other workers^18^.

The results shown in Table 3, indicate that the composition of animal faeces could substantially affect the recovery rates of the oocysts using this technique. Strikingly, the recoveries were extremely different. Therefore, it is desirable to avoid mixing the faecal and food during the collection of faeces. The nature and composition of samples influence on the performance of solutions and the effectiveness of oocyst recovery. It is clear from our results that the composition of seeded materials masks the presence of oocysts in some way. The most likely causes of this masking are the hydrophobic and surface charge characteristics, which are influenced by the ionic strength and pH of the suspension medium. They determine the degree of the adsorption of oocysts to particulate material. These results match those observed in earlier studies^25^,^26^,^27^,^28^,^29^. They reported that the oocyst experimental recovery may be affected by the nature and type of the sample, type of solutions and technique applied in oocyst concentration and purification.

The technique described above in which oocysts and eggs can be freed of faecal debris by a sugar floatation method cannot be recommended if the faecal material quantity is greater than 0.6 and 1.2 g for classical and alternative gradient centrifugation, respectively. Nonetheless, the cream separator method described herein handles large quantities of faecal material up to 960 g per run and reduces the crucial laboratory chore burden.

The proportion of sporulated oocysts was determined microscopically, and the sporulation rate of *E. mitis* was approximately 68%; however, the hatchability rate of nematode eggs was roughly 74% using the novel approach. These results are in accordance with those of refs 30 and 31, who show that sporulation under optimal condition results in 68% sporulation ability of *Eimeria acervulina* oocysts and 62% of *Eimeria maxima* oocysts, respectively. In comparison to the traditional method, no significant differences were found. Moreover, for a higher sporulation and hatching percentage of oocyst and egg population in faeces, faeces for oocyst and egg isolation may be collected in pans filled with 2.5% K_2_Cr_2_O_7_ and water, respectively.

The quality of purified oocysts and eggs refers to the degree of contaminants, including microbial and faecal debris that is present after oocyst and egg purification. The coccidian oocysts and trichostrongylid eggs were not existed in a free form; however, probably adhered to faecal debris. Furthermore, the microbial contamination was always observed in oocysts and eggs isolated with traditional methods. Sodium hypochlorite has been used successfully at a stage subsequent to centrifugal flotation (i.e. washing under vacuum) to clean faecal solids and microbial contamination from coccidian oocysts and eggs. Results similar to ours have been reported by^32^,^33^ for coccidian oocysts. Intriguingly, the current approach has been applied to explore the *H. contortus* egg microbiome (unpublished data).

In spite of the existence of several methods for egg purification, such as sucrose and lymphocyte separation medium (LSM) for *Ostertagia* egg enrichment^13^, or both salt and sugar solutions for gastro-intestinal nematode egg isolation from faeces^12^, but none has yet achieved general acceptance. The expenditure, time-consuming nature, need for particular facilities and eminently trained personnel, and large volume of environmental samples have limited the scope of using the traditional assays.

The advantages of the cream separator machine and subsequent vacuum filtration can be listed as follows: it is rapid and more productive, which will result in decision making to control the infection in the shortest duration; it can be used for small- and large-scale applications, which are required for improving our understanding of the epidemiology and genetics of host-parasite interactions; it avoids more exposure to salts, which kill the parasites in conventional salt floatation methods; and it holds the potential to explore the microbial community that may be associated with coccidian oocysts and nematode eggs.

In conclusion, these data indicate that the NaCl cream separator flotation coupled with electrical vacuum is suitable for routine detection, obtaining highly purified oocysts and eggs from faeces or food-faecal material mixtures. Moreover, the current method described here provides an alternative way to harvest and purify parasite oocysts and eggs on a large-scale and can also be scaled upwards to handle even larger amounts. Future application of the fully automatic centrifugal cream separator with mechanical washing and discharge of faecal residue could be the top-first in the field of parasitology.

## Additional Information

**How to cite this article**: El-Ashram, S. and Suo, X. Electrical cream separator coupled with vacuum filtration for the purification of eimerian oocysts and trichostronglyid eggs. *Sci. Rep.* **7**, 43346; doi: 10.1038/srep43346 (2017).

**Publisher's note:** Springer Nature remains neutral with regard to jurisdictional claims in published maps and institutional affiliations.

## Figures and Tables

**Table 1:** Summary of concentrated and recovered oocysts and their corresponding dilution factor employing chicken fecal materials (experimental infection).

Dilution factor	Percentage of oocyst recovery rate	
	Lighter layer^a^	Heavier layer^a^
1:20	15.86% ± 1.114%	0.625% ± 0.094%
1:15	44.49% ± 1.14%	7.41% ± 0.94%
1:12.5	80.68% ± 1.613%	5.29% ± 2.92%
1:10	66.47% ± 2.76%	0.19% ± 0.0497%
1:9	51.06% ± 1.579%	2.948% ± 1.296%
1:7.5	46.646% ± 3.189%	39.22% ± 2.099%
1:5	16.399% ± 2.037%	0.0057% ± 0.0037%

^a^Each value is a mean of ten separate runs.

**Table 2:** Comparison of cream separator and conventional sucrose method for the determination of maximal and minimal recovery rates of eimerian oocysts and nematode eggs.

Description	Cream separator method		Sucrose gradient method	
	Eimerian oocysts	Trichostronglyid eggs	Eimerian oocysts	Trichostronglyid eggs
Average maximal recovery ( ± SD)^a^	80.68% ± 1.61%	91.91% ± 1.35%	81.87% ± 9.81%	92.32% ± 3.13%
Average minimal egg recovery number ( ± SD)^a^ (3 oocysts/eggs per ml of applied faecal material suspension)	0.9% ± 0.74%	1.6% ± 0.55%	1% ± 1.03%	1.2% ± 0.8 4%
Viability rate^a^	68.06% ± 1.9%	73.74% ± 7.61%	69.75% ± 1.47%	74.164% ± 1.648%
Sample size	960 g		0.6 g (classical sucrose gradient set up) and 1.2 g (alternative sucrose gradient set up)	
Capacity	12 liter of faecal suspension (MOTOP C14-100, Ukraine)		7.5 ml of faecal suspension (classical sucrose gradient set up)/15 ml (alternative sucrose gradient set up)(Falcon™ 50 ml conical centrifuge tubes)	
Time required	7.2 min ± 0.8 min		15 min ± 2.05 min	

^a^Each value is a mean of ten separate runs.

**Table 3:** Recovery efficiency of oocysts spiked in different combinations of food and faecal materials of rabbit and chicken.

Description	Percentage of oocyst recovery rate (Lighter layer)^a^	Percentage of oocyst recovery rate (Heavier layer)^a^
Pure chicken food	63.38% ± 4.09%	0.12% ± 0.03%
Pure rabbit food	79.45% ± 1.12%	0.35% ± 0.02%
Chicken food mixed with chicken faeces (1:5)	36.11% ± 26.16%	0.31% ± 0.42%
Chicken food mixed with rabbit faeces (1:5)	37.12% ± 1.89%	0.06% ± 0.046%
Rabbit faecal material plus rabbit food (1:5)	89.46% ± 2.30%	2.21% ± 1.20%

^a^Each value is a mean of ten separate runs.
