# Corrigendum: Electrical cream separator coupled with vacuum filtration for the purification of eimerian oocysts and trichostrongylid eggs

**DOI:** 10.1038/srep45799

**Published:** 2017-04-05

**Authors:** Saeed El-Ashram, Xun Suo

Scientific Reports 7: Article number: 4334610.1038/srep43346; published online: 02 24 2017; updated: 04 05 2017

The original version of this Article contained typographical errors, where all instances of ‘trichostrongylid’ were given as ‘trichostronglyid’. This has now been corrected in the HTML and PDF versions.

